# An Anatomy of Fungal Eye: Fungal Photoreceptors and Signalling Mechanisms

**DOI:** 10.3390/jof9050591

**Published:** 2023-05-19

**Authors:** Özlem Sarikaya Bayram, Özgür Bayram

**Affiliations:** Biology Department, Maynooth University, W23 F2K8 Maynooth, Co. Kildare, Ireland

**Keywords:** photoreceptors, fungi, phytochromes, white collars, red light, blue light, fungal photoresponses

## Abstract

Organisms have developed different features to capture or sense sunlight. Vertebrates have evolved specialized organs (eyes) which contain a variety of photosensor cells that help them to see the light to aid orientation. Opsins are major photoreceptors found in the vertebrate eye. Fungi, with more than five million estimated members, represent an important clade of living organisms which have important functions for the sustainability of life on our planet. Light signalling regulates a range of developmental and metabolic processes including asexual sporulation, sexual fruit body formation, pigment and carotenoid production and even production of secondary metabolites. Fungi have adopted three groups of photoreceptors: (I) blue light receptors, White Collars, vivid, cryptochromes, blue F proteins and DNA photolyases, (II) red light sensors, phytochromes and (III) green light sensors and microbial rhodopsins. Most mechanistic data were elucidated on the roles of the White Collar Complex (WCC) and the phytochromes in the fungal kingdom. The WCC acts as both photoreceptor and transcription factor by binding to target genes, whereas the phytochrome initiates a cascade of signalling by using mitogen-activated protein kinases to elicit its cellular responses. Although the mechanism of photoreception has been studied in great detail, fungal photoreception has not been compared with vertebrate vision. Therefore, this review will mainly focus on mechanistic findings derived from two model organisms, namely *Aspergillus nidulans* and *Neurospora crassa* and comparison of some mechanisms with vertebrate vision. Our focus will be on the way light signalling is translated into changes in gene expression, which influences morphogenesis and metabolism in fungi.

## 1. Introduction

Life on Earth thrives with solar energy. Photosynthetic organisms such as vascular plants, microscopic and macroscopic algae and certain group of bacteria harvest the energy of sunlight and store it as chemical bond energy by synthesizing carbon-rich polymers (sugar molecules) using carbon dioxide and water molecules [1]. This energy conversion is critical for the sustainability of life on our planet as these energy-rich molecules are consumed by all heterotrophic organisms such as animals, bacteria, protozoa and fungi.

Light is one of the key signals which organisms use to orient themselves in order to survive. Generally, the visible light spectrum represents beams of light which the human eye can view or sense. Visible solar light contains seven different wavelengths from violet (350–400 nm), indigo (400–450 nm), blue (450–495 nm), green (490–570 nm), yellow (570–600 nm), orange (600–620 nm) to red light (620–750 nm). In addition to these, the far ends of visible light contain ultraviolet (UVC 200–280 nm, UVB 280–315 nm, UVA 315–400 nm) and infrared (IR 780 nm–1000 µm) light spectra, which can also be sensed by some organisms using special forms of photoreceptors. These extreme spectra, particularly UVC and UVB light, pose great risk to all kinds of life forms, as they cause production of reactive oxygen species and damage the genetic material DNA by forming pyrimidine dimers [2]. Therefore, seeing the light is necessary not only for orientation, but also for preparation for stress conditions.

Fungi represent one of the most diverse life forms, ranging from unicellular yeast to multicellular filamentous and mushroom fungi. These versatile organisms are friends and foes of mankind since they are used as food sources, food additives, fermentation agents, producers of enzymes and sources of bioactive compounds and they are plant, animal and human pathogens [3]. Fungi play important roles in sustainability of global resources by contributing to nutrient recycling and forming mycorrhiza with forest components.

Light signals help fungi to regulate their morphology and physiology. For example, growth, metabolism, development, germination, sexual fruit body formation and production of secondary metabolites and even pathogenicity can be controlled by illumination conditions [4,5,6,7,8,9,10,11,12]. Some fungi such as *Phycomyces blakesleeanus* even show a clear phototropism towards light, which is very characteristic of light response of plants [13]. Certain fungal light responses such as phototropism or fruit body formation are very overt. However, some responses such as changing metabolism require more investigation to distinguish slight effects of light on these processes. The fungal eye is much more sensitive to low light intensity and even the light intensities which a human eye cannot detect (10^−9^ mol of photons m^−2^ can be sensed by fungal eye). Since early studies of fungal light responses, the main phenomena researchers investigated was the perception of light signals by fungi and their translation into morphological, physiological, and metabolic responses. Photoreceptors are protein molecules that capture photons using their light-absorbing organic chemical groups named chromophores [14]. Absorption of photons by the chromophore generates conformational changes in the photoreceptor protein, turning it into a signalling molecule which initiates a signal transduction towards the nucleus [15].

Although the studies on physiological, physical, molecular biology and biochemical aspects of fungal light reception and signalling shed light on tremendous amount of information over the last century, light sensing and light signalling are multidimensional processes, which require further mechanistic studies. Many genetic studies, morphological and physiological observations were carried out in different plant and human pathogenic fungi. Most mechanistic knowledge comes from a few filamentous fungi such as *Aspergillus nidulans* and *Neurospora crassa*. However, *Phycomyces, Trichoderma*, *Fusarium* and *Botrytis* species have also been studied to a certain extent. Results of these studies provide some mechanistic and open-ended models. Since fungal light perception has been extensively reviewed in recent years [5,9,12,16,17,18,19], we mainly focus on molecular mechanistic models of light signalling in two model filamentous fungi, namely *A. nidulans* and *N. crassa*.

## 2. Fungal Light Responses

**Figure 1:**
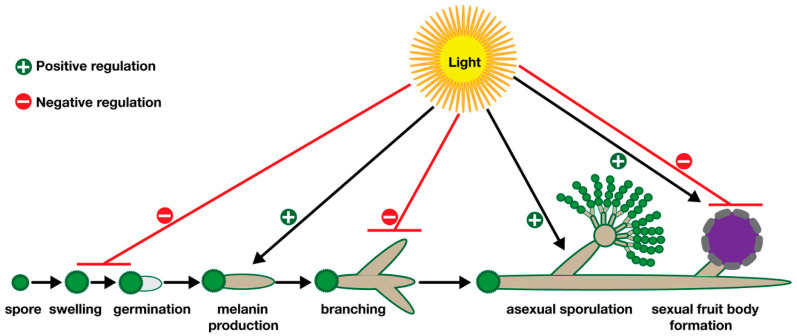
Influence of illumination on different stages of fungal development. Spores germinate by taking up nutrients and form germ tubes. Growing hyphae produce pigments (melanin or carotenes) which protect them from UV irradiation. Further growth of the hyphae leads to either asexual structures or sexual fruit body formation. Light acts as either positive or negative regulator as indicated by black or red lines.

Fungi establish different life forms throughout their life cycle. It often starts with germination of a spore which can be either an asexual or a sexual spore depending on the fungal species. Spores swell by taking up nutrients and lead to formation of germlings which further utilize nutrients and develop web-like mycelia which represent fungal vegetative life forms (Figure 1). These highly branched mycelia can differentiate into asexual or sexual organs, which finally lead to asexual or sexual spores. Along with these developmental processes, fungi also coordinate their primary and secondary metabolism [11,20] whereby production of certain pigments and bioactive metabolites is also associated with development [21,22].

Undoubtedly, light has a big impact on all these developmental and metabolic processes. For example, light often has a negative impact on germination of fungal spores which causes delays in the germination process. Spore germinations of most Aspergilli, such as the model organism *A. nidulans*, human pathogen *Aspergillus fumigatus*, and even some industrial strains of *Aspergillus oryzae*, are delayed or reduced by extended illumination (Figure 1) [17,23,24,25]. This phenomenon is not only specific to Aspergilli, but can also be seen in other genera including *Fusarium* [23], not only at the germination stage; elongation and extension of hyphae are also highly responsive to illumination (Figure 1). While light suppresses hyphal growth in *N. crassa*, on the other hand, it promotes carotenogenesis [5]. Likewise, hyphal growth of biocontrol agent *Trichoderma atroviride* is also negatively influenced by light exposure [26].

Fungi produce different pigments such as carotenoids and melanin whose biosynthesis is triggered by a light signal. These pigments are a part of an important protection mechanism against the harmful effects of UV light. They often absorb excessive energy of light and quench reactive oxygen species formed under light. Promotion of carotenoid and melanin formation by light have been shown in a range of organisms, including *Neurospora, Fusarium*, *Aspergillus* and *Phycomyces* [27,28].

The switch from vegetative growth to asexual or sexual development is also highly responsive to light and controlled by illumination regimes (Figure 1). Although light inhibits initial germination of spores, light often promotes asexual spores (conidiogenesis) in most fungi such as *Aspergillus*, *Neurospora*, *Trichoderma*, and *Fusarium*, whereas production of sexual fruit bodies of some Mucorales and Basidiomycota members also requires light [13,29]. Some other fungi such as *A. nidulans* need darkness for fruit body formation (Figure 1) [11,30].

In addition to developmental decisions, light also controls production of secondary metabolites. For example, production of the carcinogenic polyketide mycotoxin sterigmatocystin is inhibited by light exposure in *A. nidulans*, suggesting that from morphogenesis to metabolism, light signalling plays important roles in fungal world [21].

Why have all these fungal species adopted light-dependent development? Although light effects on development seem to be complicated as light inhibits spore germination while it promotes production of spores, there is a fundamental logic behind these phenomena. One of the fundamental functions of fungal photoreception is self-defense against the harmful effects of UV light. Fungal spores are densely packed with protective pigments and osmolytes which protect the next generation of fungi from stressors such as UV light and oxidative stress [31,32,33]. The second fundamental function of photoreception is possibly to find optimum conditions or time for spore dispersal [34,35]. Asexual sporulation is a default mode of sporulation in fungi, which is important for the survival of a species among competitors, which requires less energy than sexual sporulation [36]. Fungi often live in soil which is covered by organic matter preventing spore dispersal. Prior to sporulation, fungal mycelia show a positive phototropism by growing its spore-carrying structures towards the air, and light induces production of sporulation structures, which allows the fungus to initiate its default reproduction mode in order to facilitate the dispersal of spores under good weather [35]. However, this is only one side of the story since there are other responses which cannot be explained in simple ecological and evolutionary models. Therefore, these fungal responses require more detailed studies with their ecological explanations.

## 3. Fungal Photoreceptors

Photoreceptors are specialized proteins which are often associated with a chromophore molecule. The chromophore molecules are often heterocyclic organic molecules which are capable of capturing photons from light. Photoreceptors often have several protein domains with different functions. Generally, a domain of the photoreceptor is either attached to a chromophore or found in close proximity with the chromophore which, upon illumination, can establish a chemical bond to that domain. In some receptors (e.g., phytochromes), this domain receives the light signal which leads to conformational changes in protein (e.g., phosphorylation of a transduction domain) which relays the phosphate molecule to downstream proteins. On the other hand, in other kinds of photoreceptors (e.g., White Collars), conformational change triggers dimerization of the photoreceptors and activation of their transcriptional functions.

**Figure 2:**
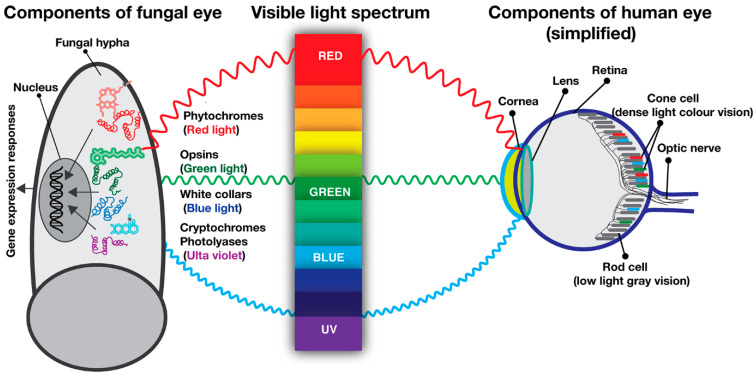
A simplified comparative depiction of main light receptors in fungi versus vertebrate (human) photoreceptor cells within the retina. A fungal cell contains three sets of light receptors, red light receptors (phytochromes), green light receptors (opsins) and blue light receptors (White Collars, vivid, cryptochromes and photolyases). Chromophores (accessory molecules) help the light receptors to capture light waves. The inner layer of the human eye, retina, contains roughly 100 million photoreceptor cells, 95% of which belong to rod cells (providing grey vision) and 4–5% belong to cone cells (providing colour vision). Rod and cone cells transmit the signals to a vision centre in the brain via optic nerves.

The fungal eye operates by using three different light receptors: blue light receptors (White Collars, vivid, cryptochromes, photolyases), green light receptors (fungal opsins) and red light receptors (phytochromes), which sense short, mid- and long wavelengths of light (Figure 2). In contrast to fungi which operate with three kinds of photoreceptors, the highly sophisticated vertebrate eye, including the human eye, uses only one kind of photoreceptor which is an opsin, green light receptor. Human eyes contain around 100 million specialized cells that can receive the light signal and convert it into neuronal impulses which are transmitted to the visual cortex of brain via optic nerves (Figure 2) [37]. There are two main types of photoreceptor cells on the surface of retina, rods and cones, respectively [38]. The rods are highly elongated cells which are sensitive to dim light and dark and contain rhodopsin as a light receptor. The rod cells make more than 90% of photoreceptor cells. They are not sensitive to colour, therefore they provide grey vision image in the brain. In contrast to the rods, the cone cells are shorter cells which contain cone opsins as light receptor and provide colour vision. Although all cone cells contain opsins, their spectral properties are different than rhodopsin and therefore they are sensitive to blue, green and red light wavelengths (Figure 2). These three types of red, green and blue cones constitute 4–5% of the total photoreceptor cell population. Combinations of these different signals (grey and colour photoreception) in the brain generate colour images by overlapping rod and cone signals. In addition to cones and rods, the retina also contains intrinsically photosensitive retinal ganglion cells (ipRGCs) which are involved in non-image forming vision that includes contrast sensing and entrainment of circadian clock [39].

As mentioned earlier, fungi use three kinds of light receptors. However, only the function of blue and red light receptors has been examined in detail. Although green light opsins have been studied to some extent, their role as light receptors is still cryptic.

## 4. Blue Light Receptors, White Collars, Vivid, Envoy, Cryptochrome/Photolyase Family

**Figure 3:**
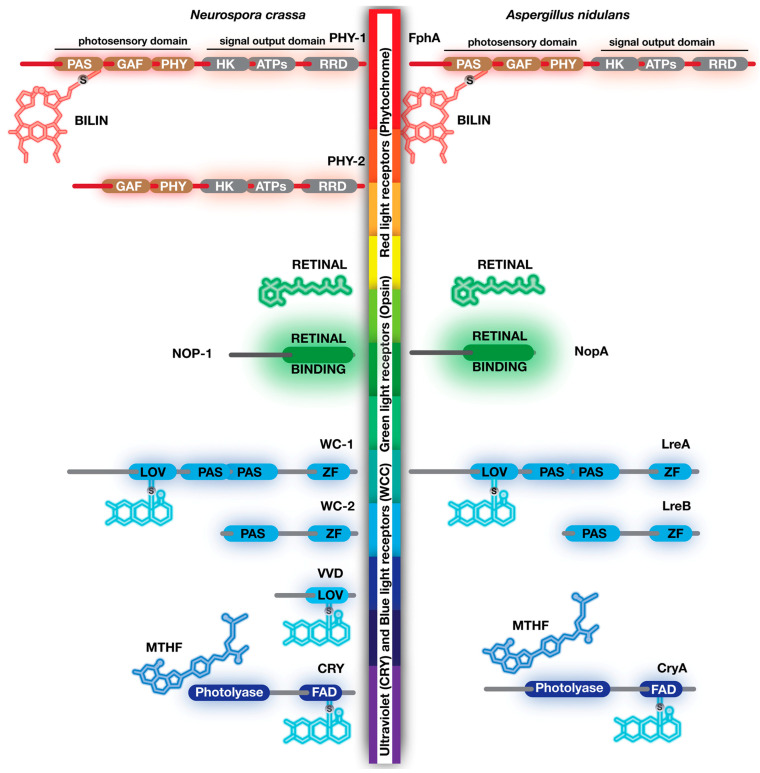
Common photoreceptor proteins and their chromophores (light-absorbing chemical groups) in two model filamentous fungi *N. crassa* and *A. nidulans*. Phytochromes, PHY-1 and PHY-2 in *N. crassa* and FphA in *A. nidulans* are light receptors that are attached to a linear tetrapyrrole (bilin) through a cysteine residue on PAS domain. The N-terminus of phytochromes acts as photosensor and contains three domains, PAS, GAF (cGMP-Adenylyl cyclase-FhlA) and PHY (distantly related to PAS) domains. The C-terminus of phytochromes contains signal transduction features with HK (histidine kinase), ATPase and RR (response regulator) domains which are similar to a hybrid form of bacterial two-component system. Red light causes conformational change on bilin and switches it from red-light-absorbing (Pr) to far-red-absorbing form (Pfr). Transmembrane fungal opsins are associated with retinal, an aldehyde form of vitamin A. Green light triggers conversion of 11-cis retinal to all-trans retinal. Blue light receptors are White Collar proteins (WC-1 and WC-2 in *N. crassa*, LreA and LreB in *A. nidulans*), vivid (VVD) and cryptochromes CRY (*N. crassa*) and CryA (*A. nidulans*). LOV (light oxygen voltage) domains found in WC and VVD or Flavin Binding (FBD) domain found in cryptochrome family are attached to FAD (flavin adenine dinucleotide) via a cysteine residue in these domains. Cryptochrome or photolyase family might have an additional accessory molecule, MTHF (methyl–tetrahydrofolate), which is not covalently attached to these proteins but found to be associated with them. In addition to the LOV domain, White Collar proteins also contain PAS (Per–ARNT–Sim) that mediates protein–protein interactions and a zinc finger DNA-binding domain which can bind to target sequences. The size of each protein is arbitrarily proportional to their amino acid sequence length (not shown).

Blue light receptors are historically the first and most-studied light receptors. Although there are four kinds of blue light receptor families, White Collars, vivid, Blue F protein and cryptochrome/photolyase, respectively, only White Collars and cryptochrome family are discussed here due to their well-conserved nature. The vivid protein is also mentioned due its role in resetting the White Collar-mediated light signalling. White Collar proteins are the most thoroughly studied fungal light receptors, which were initially studied in *N. crassa* [5,40,41]. They were named after the *wc-1* and *wc-2* mutants which could not photoinduce carotenogenesis [40]. There are often two WC proteins encoded in fungal genomes with some exceptions, for example, that very few fungi might have more than three copies of *wc* encoding genes [10,42]. There are two WC protein homologs (WC-1 and WC-2) in both *N. crassa* and (LreA and LreB) *A. nidulans* (Figure 3). Three Per Arnt Sim (PAS) domains are present in WC-1 and LreA [43,44,45]. Generally, PAS domains are known for mediating protein–protein interactions [43]. However, the first one of the PAS domains in WC-1 has evolved as a light oxygen voltage (LOV) domain. Flavin adenine dinucleotide (FAD) acts as a chromophore for blue light sensing. Upon illumination with blue light, a highly reactive cysteine residue in LOV domain makes a covalent FAD-thiol adduct [46]. Keeping the protein in the dark reverses this biochemical reaction, leading to cleavage of the covalent bond between LOV domain and FAD, which is named a photocycle. The remaining PAS domains mediate interactions of WC-1 with other proteins such as WC-2. In addition to LOV and PAS, WC-1 (LreA) also contains a GATA-type zinc finger motif, which mediates DNA binding. WC-2, which is a shortened version of WC-1, lacks LOV and one PAS domain. WC-1 and WC-2 form a White Collar complex (WCC) heterodimer which is capable of binding to DNA and control gene expression [47]. Although the genomes of *N. crassa* and *A. nidulans* contain two WC homolog proteins, some other fungal genomes encode more WC homolog proteins as can be seen in *Mucor* and *Phycomyces* where they regulate various light responses, including carotenogenesis and phototropism [48,49].

In addition to WC proteins, the second group of blue light photoreceptors is vivid protein (VVD), which is not widely distributed in the fungal kingdom (Figure 3). Only very few fungi have VVD homologs, such as *Neurospora* and *Trichoderma*, where they are also called ENVOY [50,51,52]. Both proteins are capable of binding FAD or FMN depending on the amino acid compositions. The function of VVD is to regulate gene expression in response to light intensity, and VVD resets gene expression under constant illumination, which is also termed photoadaptation. VVD proteins can be dimerized and interact with the WCC complex, which desensitizes WCC-dependent light responses, thereby allowing rebinding of new WCC complexes to light-responsive promoters [53,54].

The third group of blue light receptors in fungi are the cryptochrome/photolyase family proteins, whose function is still not very well understood in comparison to WCC and VVD. A founding member of this group is actually a DNA repair enzyme. The DNA photolyases repair pyrimidine dimers formed as a result of UV radiation [55]. Photolyases are activated upon illumination with UVA light and break pyrimidine dimers. Cryptochrome proteins have evolved from DNA photolyase family proteins which have ultimately lost their DNA repair function but gained regulatory roles for gene expression [56]. There are still hybrid cryptochromes which can affect both gene regulation and DNA repair. However, they show their repair activity in single-stranded DNA [57]. They are evolutionary transition forms between photolyases and cryptochromes, which are collectively named a Cry-DASH family. Cryptochrome family proteins have a FAD-binding domain. However, in contrast to WC and VVD proteins, cryptochromes bind FAD or methyltetrahydrofolate (MTHF) without a covalent bond. A cyclobutane pyrimidine dimer (CPD) photolyase CryA has been shown to be involved in DNA repair and regulation of sexual fruit body formation in *A. nidulans* [58]. Very recently, CryA was proposed to regulate some phytochrome-dependent gene expression in various illumination regimes in *A. nidulans* [59], whereas *N. crassa* CRY protein belongs to the Cry-DASH family, and the heterologously-expressed form of CRY can bind to FAD and MTHF. However, transcriptome studies suggest that it has a fine-tuning role in light-dependent gene expression [60,61].

## 5. Green Light Receptors, Opsin Family

Opsins are membrane-bound proteins with seven transmembrane helices which can bind to retinal and act as a light receptor for green light. They are the main components of the vertebrate vision, including the human eye. They are found in the membrane discs of rod and cone cells where they act as initial light receptors. However, their roles in fungal vision were not completely understood. Although many fungal genomes encode an opsin protein, they show similarity to bacterial and archaeal rhodopsins which often act as proton pumps rather than light receptors. Some of the fungal opsins possess lysine residue in the seventh helix which binds to retinal, whereas some do not have the lysine residue critical for retinal binding. While *N. crassa* NOP-1 has a lysine residue, *A. nidulans* and *T. atroviride* opsin proteins do not possess the lysine [62,63,64]. Some fungal opsins such as *N. crassa* NOP-1 were shown to have a slow photocycle with proton pumping activity rather than light reception. Similarly, *Fusarium* opsin CarO acts as a light-dependent proton pump controlling spore germination [23]. A very recent study has illuminated the role of green light receptors in fungi, particularly in common ancestor of the fungi. A primitive fungal eye (light sensing organ), an eyespot, also called a side-body complex, has been found to contain lipids and a very interesting fusion protein CyclOP [65]. CyclOP contains two domains, a microbial rhodopsin and guanyl cyclase enzyme domain which is the main secondary messenger in vertebrate vision cells (see next section). This organ is only found in zoosporic fungi, Chytridiomycota, which represent the early diverging fungal lineages [66]. However, this organ cannot be found in other fungal groups outside of Chytridiomycota. Although the findings from different fungi suggest that opsin family proteins act as light-dependent proton pumps, this recent evidence suggests that they may have more important roles in early diverging fungi.

## 6. Red Light Photoreceptors, Phytochromes

Phytochromes are red light receptors which contain multiple domains for light reception and signal transduction. One of the striking features of the phytochromes is that they are not found in vertebrates but are specific to sessile organisms such as plants, bacteria and fungi [9,67,68]. Although certain fungi contain phytochrome-encoding genes, most of the mechanistic studies come from model fungus *A. nidulans* where only one phytochrome protein exists [44,68]. However, *N. crassa* possess two phytochrome proteins PHY-1 and PHY-2, only one of which influences development. While the fast-evolving PHY-2 protein is important for timing of sexual fruit bodies, both PHY-1 and-2 regulate gene expression [69,70].

Phytochrome domains can be mainly divided into two parts: (I) N-terminus contains photosensory region which receives the light signal and (II) C-terminal region possesses signal transduction or signal output domain which shows protein kinase activity (Figure 3). There are three domains at the N-terminus: (I) the Per—ARNT–Sim (PAS) domain, (II) the cGMP-Adenylyl cyclase-FhlA (GAF) domain and (III) a distantly related to PAS (PHY) domain (Figure 3). Phytochromes use bilin (linear tetrapyrrole) molecules as chromophore which is autocatalytically linked to a cysteine residue in the PAS domain. Upon reception of red light, chromophore (P_r_) changes its conformation, and therefore its spectral properties. This new form of bilin can absorb far-red light (P_fr_). The two forms of bilins are interchangeable upon illumination with red or far-red light (P_r_-P_fr_).

The signal transduction part contains three domains: (I) histidine kinase (HK), (II) ATPase and (III) response regulator (RR) domains which are responsible for transmitting light signals to downstream elements. Histidine in the HK domain is phosphorylated in fungal and bacterial phytochromes, whereas plants do not possess histidine in the HK domain [71]. The presence of an RR domain in fungal phytochromes is a reminiscent of the two-component signal transduction systems from bacteria. Bacteria contain an average of 30–40 two-component systems for sensing and responding to environmental signals [72]. Often, a membrane-bound sensor kinase receives an environmental signal and autophosphorylates its histidine residue, which transfers this phosphate group to an aspartate residue of RR protein, which is the second component of the two-component system. Similar to operation of two-component system, the HK domain of the fungal phytochrome can phosphorylate the arginine of an RR domain which becomes active for further downstream signal transduction. In vitro studies with *A. nidulans* phytochrome FphA indicate that FphA can autophosphorylate its histidine in the HK domain, which can pass this phosphate to the aspartate residue in the RR domain [73]. Chromophore bilin and illumination are required for autophosphorylation of FphA. However, HK to RR transphosphorylation does not require chromophore, suggesting that FphA apoprotein can also perform transphosphorylation. There is one question that remains to be answered for *A. nidulans* FphA: where does the bilin come from to make a functional phytochrome? Normally, heme oxygenases synthesize the tetrapyrrole molecules. Although *A. nidulans* FphA can bind two forms of bilins in vitro, a potential heme oxygenase encoding gene could not be identified in the *A. nidulans* genome. However, in another plant, pathogenic fungus *Alternaria alternata*, deletion of two heme oxygenase genes resulted in similar phenotype to that of phytochrome deletion [74]. Furthermore, these two enzymes physically interacted with phytochrome, suggesting a potential metabolon (sequential transient multiprotein complexes that facilitate substrate processing) production. These data suggest an important role for mitochondria for assembly of phytochrome and heme oxygenase on the surface of mitochondria. It will be interesting to determine whether these two heme oxygenases are conserved in *A. nidulans* and whether they participate in production of functional phytochrome in mitochondria.

## 7. Molecular Mechanism of Light Signal Transduction

**Figure 4:**
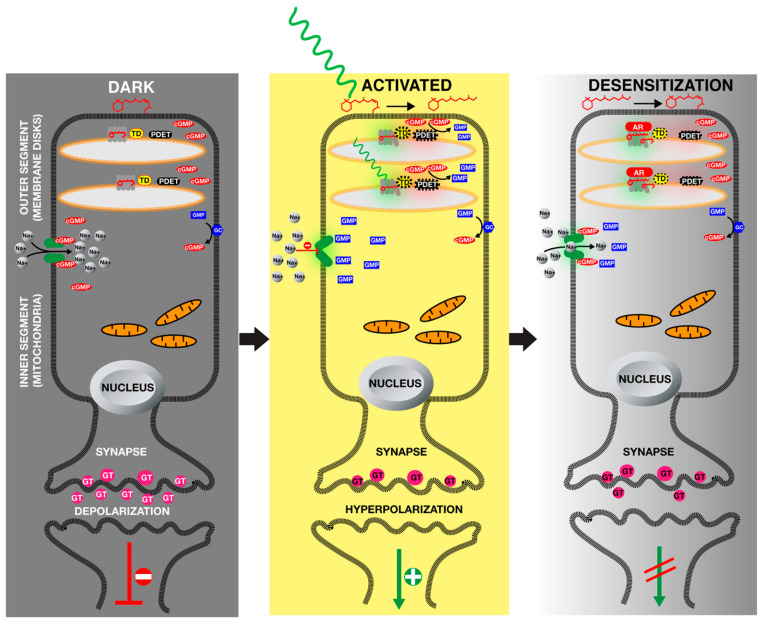
Phototransduction in rod cells in vertebrate retina. Outer segments of rod cells contain numerous membrane disks where seven transmembrane rhodopsin photoreceptors are embedded. In the dark, rhodopsin-associated retinal is in its 11-cis retinal form, which keeps heterotrimeric G protein transducin (TD) inactive, therefore phosphodiesterase (PDET) is also inactive. Membrane-bound guanyl cyclase (GC) synthesizes cyclic guanosine monophosphate (cGMP) from guanosine monophosphate (GMP). cGMP facilitates the opening of cation channels which transport sodium ions inside the rod cells, which keeps the cells in a depolarized condition leading to the release of glutamate (GT). This, in turn, keeps the vision neurons inactive. Upon reception of light, 11-cis retinal is turned into all-trans retinal and cannot associate with rhodopsin, causing conformational change in rhodopsin, which triggers TD-PDET activation. This, in turn, leads to reduced levels of cGMP because of PDET activity, which results in the closing of cation channels. This keeps sodium ions outside of the cell and therefore leads to hyperpolarization of the rod cell which cannot secrete GT into synaptic area. Therefore, vision neurons are activated. Phototransduction is desensitized by calcium signalling. During dark adaptation periods, low-level calcium activates the rhodopsin kinase which phosphorylates the rhodopsin (not shown). Finally, arrestin (AR) binds to phosphorylated rhodopsin and deactivates phototransduction.

Photoreceptors absorb light using their chromophores, which causes conformational changes in photoreceptor protein. These conformational changes may influence its stability, protein–protein interactions (dimerization, homo- and heterodimer formation or participating multimeric complexes), cellular localization, DNA-binding capability or its phosphorylation state. All these molecular events are translated into physiological, cellular and behavioural responses. Maybe one of the most striking ways that light signals turn into an image is the phototransduction which takes place in vertebrate eyes. Human eyes contain more than 100 million photoreceptor cells in the retina. Rod (grey vision) and cone (colour vision) cells provide vertebrate vision (Figure 4). The majority of vision is provided by the rod cells which are elongated neuronal cells containing hundreds of stacking membranes (discs) within the tip of cells [75]. In these discs, there are membrane-bound rhodopsins associated with green light chromophore retinal. In the dark, retinal chromophore is found in its 11-cis conformation. Two signalling proteins, membrane-bound heterotrimeric G protein, transducin and a phosphodiesterase are not active [76]. Rod cells contain enough cyclic guanosine monophosphate (cGMP) and this molecule keeps sodium channels open and transports sodium cations into the rod cells. Guanyl cyclase (GC) found in plasma membrane of rod cells constantly generates cGMP for this process, which opens cation channels that transport sodium into the rod cells, which results in depolarization of the rod cells and release of glutamate (GT) into the synaptic area. GT inhibit excitation of vision neurons (Figure 4, Dark).

Upon illumination with light, this 11-cis retinal of rhodopsin is turned into all-trans retinal which causes conformational change in the rhodopsin protein (Figure 4, Activated). The activated rhodopsin in turn switches on transducin, which directly activates phosphodiesterase that converts cGMP to GMP, therefore reducing cGMP levels within the rod cells. This low level of cGMP results in closing of cation channels preventing sodium accumulation within the cell. Low levels of sodium result in hyperpolarization of the rod cells, which prevents the release of GT. Lack of GT in the synaptic area triggers excitation of optic neurons, transferring an electric signal to the brain for vision.

To reset the photoreceptor and ensure reception of the light signal, rhodopsin is phosphorylated by rhodopsin kinases and this phosphorylated rhodopsin is bound by AR, which causes desensitization of rhodopsins, ensuring the reactivable form of phototransduction machinery (Figure 4 Desensitization).

What about fungal photosignalling mechanisms? How do they operate? Do they show similarities to vertebrate phototransduction mechanisms? If not, what are the consequences of light signalling in fungi? Thus, two well-characterized light signalling mechanisms are discussed in detail. There are numerous observations with photoreceptor mutants across the fungal kingdom. These findings have been extensively discussed in excellent reviews [5,9,16,17,28,77,78]. Therefore, rather than observations, we focus on only mechanistic and solid data originating from several model systems, which mainly concentrate on blue and red light sensing. Furthermore, we compare and contrast similar characteristics of the fungal eye with vertebrate vision although both systems have evolved differently by developing different light receptors and using them for different purposes.

## 8. Blue Light Signalling, Regulation of Light-Responsive Genes by White Collar Complexes

**Figure 5:**
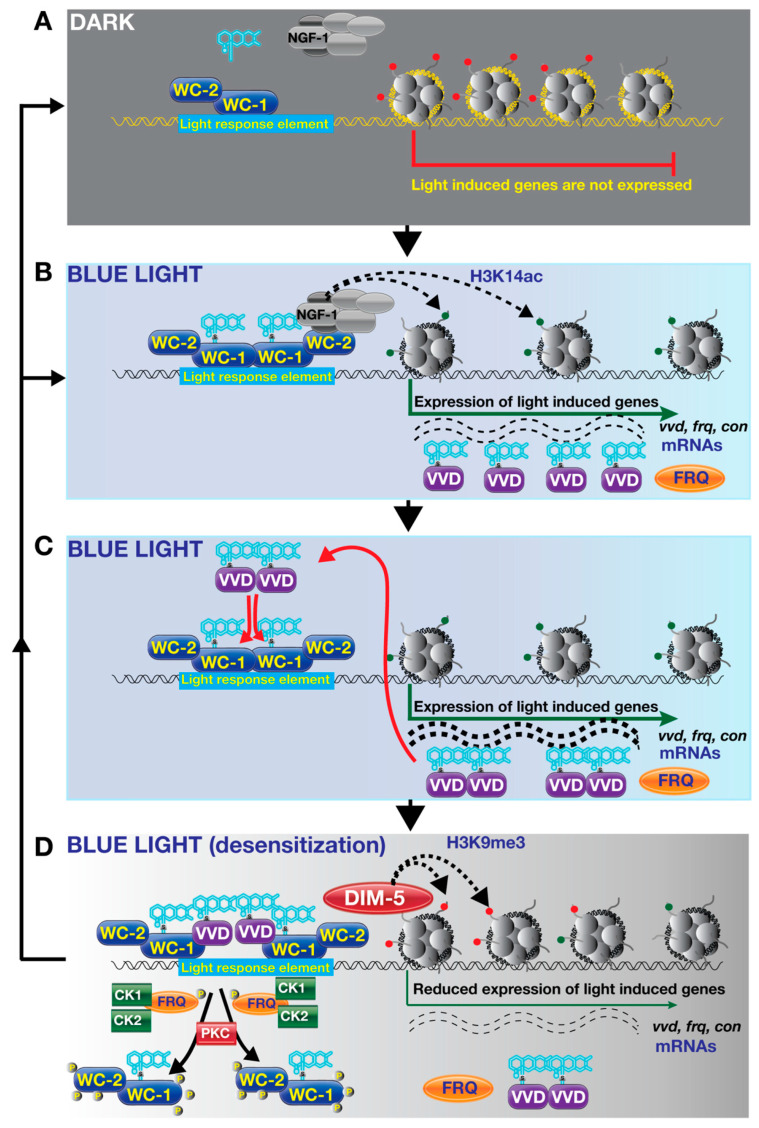
Blue light signalling and its consequences on gene expression. (**A**) Dark condition: The WC-1 and WC-2 form a WCC complex and bind to light-response elements in the promoters of light-regulated genes in the absence of light. Light-responsive genes presumably have H3K9 methylation or similar, which results in gene silencing. (**B**) Upon blue light exposure, FAD is attached to LOV domains of WC-1, which induces heterodimer formation of the WCC heterodimers. This also induces histone acetyl transferase NGF-1-dependent acetylation of nucleosomes and gene expression. (**C**) Light-induced genes, including conidiation genes, photoadaptation gene *vvd* and frequency gene *frq*, are transcribed by transcription machinery. VVD proteins, which are translated from *vvd* mRNA, form homodimers under illumination via covalent bonding to FAD. *frq* mRNA is also translated to the FRQ protein. (**D**) These VVD homodimers interfere with the WCC complexes via interacting with the WC-1 subunit. This reduces binding efficiency of the WCC complex to target promoters, which diminishes light-dependent gene expression. Further displacement of WCC from promoters is catalyzed by protein kinase C (PKC) and FRQ-associated kinases, casein kinase 1 and 2. Displacement of WCC is further mutually influenced through the methylation of Histone 3 Lysine 9 by histone methyltransferase, DIM-5. This ensures reactivation of blue light signalling for another round.

White Collar proteins, WC-1 and WC-2, are two blue light receptors which are the best characterized photoreceptors in the fungal kingdom. They provide invaluable insights into light signalling and molecular regulation of circadian rhythms in eukaryotes [79]. Their role in circadian rhythms has been discussed by many elegant reviews [28,77,80,81,82,83]. In this section, we mainly focus on their photoreceptor function rather than their roles in the circadian cycle. One of the important features of the WC system is that WC-1 is a photoreceptor, whereas WC-2 is not a photoreceptor, but both are transcription factors since they contain GATA-type zinc finger domains. This makes them direct signal transduction proteins for light signalling. Although many proteins regulate their activity, such as VVD, CK-1, FRQ and FRH, they nevertheless do not need other signalling proteins between light signal and DNA-dependent responses. We exemplify that the WCC complex turns light signalling into gene expression and physiological responses in Figure 5.

For DNA-binding proteins, it is important whether they bind to DNA transiently in response to a signal or are continuously tightly bound to DNA. The WC-1 and WC-2 proteins are capable of binding to light-response elements of the early light responsive genes via their zinc finger domains both in the dark and light (Figure 5A,B) [47,84,85,86,87]. As mentioned earlier, blue light causes covalent bond formation between the cofactor FAD and the LOV domain of WC-1, which also results in heterodimer formation of WC-1 and WC-2 (Figure 5B). Does WCC directly regulate transcription? It was shown that WC-1 interacts with NGF-1, a subunit of SAGA/ADA histone acetyl transferase complex. Promoters bound by WCC complex show NFG-1-dependent acetylation suggesting the activation of early-light-responsive genes by chromatin remodelling induced by Histone 3 Lysine 14 acetylation (H3K4ac) [88]. It is interesting that in *A. nidulans*, WC homologs (LreA-LreB) were also found to be associated with GcnE, which is one of the subunits of the SAGA/ADA complex [89,90]. A GATA type transcription factor SUB-1 and O-acetyltransferase domain protein FF7, which are involved in light-dependent gene expression in *N. crassa*, have also been shown to depend on the WCC for chromatin remodelling including displacement of histones in light-responsive promoters [91]. mRNAs of light-regulated genes such as *vvd*, frequency (*frq*) and conidiation (*con*) genes are accumulated as a result of synergistic effect of WCC, NFG-1 and SUB-1 (Figure 5C) [40,84,85,92]. Short illumination (as little as 15 min) results in activation of about 400 light-dependent genes [91,93,94]. Light acts as a double-ended sword for the WCC complex, (I) activating the WCC-driven transcription, (II) initiating light-dependent degradation of both WC-1 and WC-2, which ensures resetting of light responses (see below). Posttranslational modifications, particularly phosphorylation, regulate activity of WC-1 and WC-2 [85,95,96]. The FRQ protein along with the FRQ-related helicase (FRH) recruits casein kinases (CK1 and 2) and facilitates phosphorylation of the WCC [97,98,99,100]. FRH stabilizes the FRQ protein, which ultimately negatively impacts the WCC activity [101]. Similarly, protein kinase A and C (PKA and PKC) interact with and phosphorylate the WCC. Furthermore, it was shown that WC-1 levels do not increase to wild-type levels in the constitutively active PKC mutant, suggesting that phosphorylation is a key process for the WCC levels [95,102,103,104].

Is there a resetting mechanism of blue light signal in fungi similar to resetting of phototransduction in rod cells where arrestin binds to rhodopsin and shuts down transduction so that the rhodopsins can receive the light signal again [105]? Not exactly, but a similar system exists in *N. crassa,* where auxiliary light receptor VVD performs this job (arrestin function), which is also called photoadaptation. Once light activates light-induced genes via the WCC complex and histone acetylation, *vvd* transcription peaks and decreases even under prolonged light conditions, finally reaching transcription levels of those in the dark. One of the striking features of *vvd* mutants is that they have a dark orange colour due to highly increased carotenoid pigments [84,106]. This is because *vvd* mutants do not show a peak-like transcription profile but demonstrate constantly increased light-dependent mRNA accumulation.

*vvd* is one of the WCC-dependent genes whose transcription depends on the light signal. Once transcribed and translated, upon illumination, VVD proteins bind FAD molecules and form homodimers via their LOV domains. These homodimers can also interact with the LOV domain of WC-1, interfering its interaction with WC-2, thereby reducing WCC levels. VVD-WC-1 interaction may stabilize WC-1 levels, which is also degraded in response to light. However, this stabilization does not contribute to WCC activity. VVD-WC-1 interaction is a key step to reset the light signal (Figure 5D) [53,107]. The function of VVD, which reduces transcriptional responses of the WCC complex by interfering WCC formation between WC-1 and WC-2, is similar to the arrestin function in the vertebrate rod cells. The function of the FRQ-associated kinases CK1 and CK2, which play important role in phosphorylation of WCC and reduce transcriptional responses, is similar to the role of rhodopsin kinases, GRK1 and GRK7 in the human eye [98,104,108]. Rhodopsin kinase phosphorylates the light-activated rhodopsins which normally bind to transducin to activate phototransduction. However, this phosphorylation facilitates binding of arrestin to the rhodopsins, which ultimately prevents activation of transducin by masking the transducin interaction site of the rhodopsin [109,110].

Like activation of light-dependent promoters, deactivation is also regulated by epigenetic markers. In *N. crassa*, silencing Histone 3 Lysine 9 trimethylation (H3K9me3) marks are catalyzed by the DIM-5 protein [111]. Photoadaptation is coordinated with histone methylation. *frq* gene is part of a circadian clock system and transcribed by WCC in response to light. Under normal conditions, *frq* mRNA, like all light-regulated mRNAs, peaks after illumination and decreases over time under constant illumination as part of the light-resetting system provided by the VVD-WC-1 interaction. However, deletion of DIM-5 (lack of H3K9me3 marks) results in more *frq* mRNA accumulation, even after prolonged illumination associated with more WCC binding in the *frq* promoter. It will be interesting to see how different histone modifications are distributed along the light-dependent genes during photoadaptation process. Are there other key modifications associated with active and inactive genes? Answering this question will further deepen understanding of light-dependent activation of genes.

**Figure 6:**
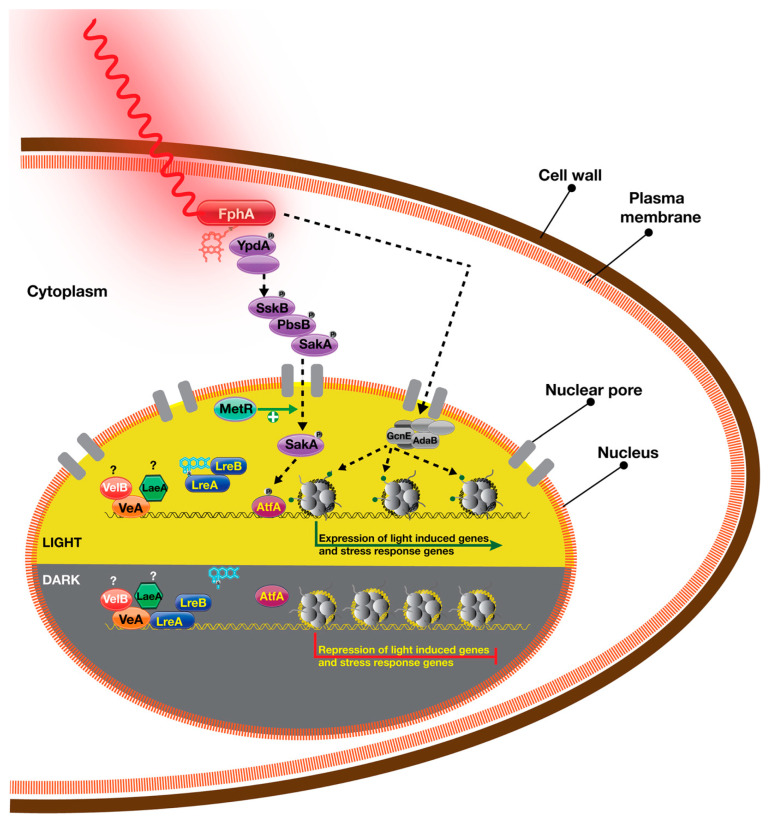
Red light signalling and its influence on gene expression in the light and dark. The bilin chromophore captures red light, resulting in a conformational change at its C-terminal region. It interacts with a phosphotransferase protein YpdA, causing its dephosphorylation. This finally results in the activation of high osmolarity glycerol (HOG) pathway by phosphorylating three mitogen-activated protein kinases (MAPK). Stress response kinase SakA enters into nucleus where it phosphorylates transcription factor AtfA that facilitates transcription of light-induced and stress-induced genes. MetR facilitates nuclear enrichment of SakA. Phytochrome interacts with two subunits of SAGA-ADA complex, GcnE and AdaB, which presumably promotes Histone 3 Lysine 9 acetylation (H3K9ac). LreA (WC-1 homolog) potentially binds to light-induced promoters but dissociates from these promoters upon light exposure. It is not known if phytochrome-induced signalling displaces LreA from these promoters. A subunit of the light-dependent heterotrimeric transcriptional complex, VeA, also binds to light-induced promoters. It is likely that VeA binds to these promoters as a heterotrimeric complex. In the dark. VeA, potentially together with VelB and LaeA, can bind to light-induced promoters. LreA (potentially together with LreB) can also bind to light-induced promoters in the dark. LreA presumably inhibits SAGA-ADA complex via its interaction with the GcnE subunit.

Many homologs of WC-1 and WC-2 have been studied in other fungi at the genetic level. However, the only mechanistic data in addition to *N. crassa* come from *A. nidulans*, where LreA (WC-1) and LreB (WC-2) bind to promoters in the dark and upon illumination LreA-LreB disengage from light-dependent promoters (Figure 6), which is different from the WCC actions in *N. crassa* [89]. This could be due to red light receptor phytochrome, which governs most of the light responses in *A. nidulans.* However, these dynamics require further genome-wide ChIP-seq studies to understand how the LreA-LreB complex acts along the fungal chromosomes. Particularly, performing ChIP-seq after dark incubation and short and long time periods might lead to dynamic data, which can further explain this opposite roles of *A. nidulans* WC homologs in this fungus.

These invaluable data, and the model drawn based on these findings, suggest that blue light signalling is a highly sophisticated signalling mechanism which has evolved in fungi to control light-dependent gene expression. One of the striking and common features of blue light signalling in fungi and phototransduction in vertebrate eyes is their resetting capacity which allows the photoreceptors to respond to a new set of photons once previous signals cause cellular responses.

## 9. Red Light Signalling, Control of Light-Responsive Genes by Phytochrome Mediated Signalling Mechanisms

One of the important differences between red and blue light signalling is that the blue light signalling protein WC-1 has both a light-sensing (FAD adduct formation) and a DNA-binding domain (ZF). This makes blue light signalling very robust and a direct regulator with respect to phytochrome-mediated red light signalling which uses downstream signalling cascade. Red light signalling has not been studied as extensively as blue light signalling in fungi. The molecular mechanism of red light signalling has been studied in *A. nidulans* since the initial discovery of phytochrome in this fungus in 2005 [68]. However, the findings regarding red light sensing provide different mechanisms from the blue light sensing phenomena. On the other hand, recent findings suggest that phytochrome-mediated red light signalling utilizes a sophisticated signal relay mechanism involving stress response signalling cascades [112]. Although *A. nidulans* phytochrome FphA contains nuclear localization signals and interacts with LreA/LreB (the WCC complex ortholog) and the velvet family regulator VeA in the nucleus, its localization to the nucleus in a light-dependent or independent fashion has not been documented yet [89]. In contrast, plant phytochromes can shuttle into the nucleus in response to illumination [113]. Cellular localization of FphA fused to GFP is generally found in cytoplasm and the nuclei are devoid of the FphA-GFP signal [68]. It remains unclear under which condition FphA is imported into nucleus or how FphA interacts with VeA and LreA-LreB in the nucleus. It is possible that a tiny fraction of active FphA might enter into the nucleus which is below the limit of detection using confocal microscopy. To prove this and understand the nuclear-cytoplasmic shuttling of FphA, nuclear enrichment studies followed by immunoblotting under red and far-red light could be useful approaches.

In *A. nidulans*, both blue and red light induce conidiogenesis. However, red light is more effective than blue light [44]. Gene expression and chromatin immunoprecipitation studies with two genes associated with conidiation, *ccgA* and *conJ*, underline two distinct functions of red and blue light sensors in *A. nidulans* [89]. In *N. crassa*, *ccgA* and *conJ* homologs are highly responsive to illumination and upregulated under blue light conditions. However, in *A. nidulans*, only red light causes upregulation of these two genes, and blue light does not cause as much of an increase as does the red light. Furthermore, only LreA and LreB bind to promoters of these genes under darkness but dissociate from the promoter under light conditions, suggesting repressor functions of LreA-LreB for these genes in the dark. However, FphA could not be immunoprecipitated in promoters of these two genes. How then does FphA activate transcription of these genes if red light activates their transcription and FphA does not have direct physical interaction with these promoters? Lack of FphA in these promoters does not prove that FphA does not bind them. Maybe chromatin immunoprecipitation strategies might not be sensitive enough to catch FphA enrichment in these promoters. Another potential candidate which mediates conidiogenesis is the bridging subunit of the velvet complex, VeA, since (I) it interacts with FphA and (II) it is also associated with *ccgA* and *conJ* promoters independent of light. Clock-controlled gene 1 (*ccg-1*) and conidiation gene 10 (*con10*) of *N. crassa* are homologs of *ccgA* and *conJ* in *A. nidulans*. It seems that VeA, LreA and LreB all occupy these promoters [89]. VeA could be activating these gene expression under red light since it interacts with FphA. In addition to its role in fruiting body development, VeA is also required for conidiation in *A. nidulans*. Not only VeA, but two other velvet family proteins, VelB and VosA, as well as methyltransferase LaeA are important for conidiation [114,115]. Since FphA is not associated with these promoters, it could well influence VeA and other proteins which regulate conidiogenesis in these promoters. One of the interesting findings is that VeA represses gene expression in the dark, suggesting a dual role for VeA, repressing in the dark and activating in the light in agreement with its proposed role and current models [116].

One of the striking discoveries of the FphA signalling was made in mutant screening where light-unresponsive mutants were identified. One of them was a mitogen-activated protein kinase (MAPK), SakA (HogA) which is part of high-osmolarity glycerol (HOG) stress pathway. Assuming light is not involved, this pathway is activated in response to different kinds of stress, including but not limited to starvation, osmotic and oxidative stress [117,118]. Phosphotransfer protein YpdA can initiate HOG pathway signalling via red light receptor FphA which has a phosphorylated histidine residue and an RR domain with an aspartate. It was discussed that the FphA aspartate can accept phosphate from YpdA which is important for dephosphorylation of the YpdA (activation) downstream signalling [112,119]. There is in vitro biochemical evidence of phosphotransfer from YpdA to FphA [119]. FphA is able to accept phosphate group from YpdA, which can activate the HOG pathway in an FphA-dependent manner. Furthermore, physical interaction between YpdA and FphA was shown to take place supporting all potential scenarios mentioned above. However, the exact mode of *in vivo* activation of this pathway is currently unknown. This signal relay system transmits phosphorylation signals to downstream signalling proteins including MAPK SakA, which enters the nucleus in response to light or stress. It was shown that FphA induces nuclear entry of SakA and its phosphorylation under light conditions [112]. This behaviour of SakA is not influenced by LreA-LreB, but blue light also induces SakA nuclear enrichment, suggesting some functional connection to unknown blue light-driven factor. SakA phosphorylates the specific transcription factor AtfA which activates gene expression, because it was shown that expression of *ccgA* and *conJ* could not be induced in mutants of the HOG pathway including *sakA*, *atfA*, *sskA*, *sskB* and *pbsB* deletions. This model suggests that stress response signalling pathways receive inputs from FphA-driven red light sensing. It will be interesting to learn if FphA only operates over the HOG pathway of its light-driven functions or uses other signalling modules such as pheromone response MAPK signalling pathway.

Recent gene expression data from *A. nidulans* suggest that there are at least 100 genes regulated by red light in the absence of FphA [59]. Furthermore, there are also blue light-induced genes in the absence of LreA protein. As an additional player, CryA has been proposed to regulate some of these genes. However, both intriguing data suggest that there might be light-sensing proteins or signalling pathways other than FphA and LreA-LreB systems. MetR, a yeast homolog of Met30 transcription factor, which is a regulator of sulphur metabolism, is a potential candidate for phytochrome-meditated light responses [120]. MetR deficiency is identical to the *fphA* deletion phenotype. Furthermore, in the absence of MetR, similar to the absence of FphA, SakA could not be properly localized to the nucleus under stress conditions (Figure 6). However, it is currently unknown how MetR and FphA interact in light signalling at a molecular level.

## 10. Conclusions

Transduction of light signals both at the receptor and downstream regulatory protein levels has been well studied in a number of model organisms including *N. crassa* and *A. nidulans*. Data coming from plant and human pathogenic fungi such as *Fusarium*, *Botrytis* and *A. fumigatus* on light regulation have further supported and diversified the established models. White Collar proteins have contributed to the understanding of the light/blue regulation in *Neurospora*, which also led to the discovery of similar light receptors in other agriculturally and medically important fungi. The discovery of red light sensing and phytochrome systems in another important fungus *A. nidulans* along with *N. crassa* opened up a different regulatory mechanism. Although we understand the main regulatory mechanisms behind light signalling, we still need to understand light regulation in other plant and human pathogenic fungi in greater detail. In particular, protein immunoprecipitation experiments coupled with mass spectrometry can help to identify key proteins that interact with the light receptors. In addition to the protein–protein interaction studies, further genome-wide binding studies coupled with next generation sequencing for those receptors binding to DNA such WC homologs can reveal mechanisms of activation of gene expression in these fungi in response to host factors and light.

We are entering a sustainability era where we need to protect and conserve the resources of our planet responsibly. In this respect, fungi position themselves at the centre of sustainability as nutrient cyclers, future food sources, enzyme and bioactive drug sources, animal leather replacements and even construction materials for the building industry [121,122]. To be able to use fungi for sustainable purposes, we need to understand how light influences their metabolism, enzyme production and biomass. If we understand the positive influence of light regimes on beneficial characteristics of fungi, we can exploit these promising paths even further. More mechanistic and omics studies of light signalling within fungi will help us to responsibly use these enormously versatile organisms for the future of our world in a sustainable manner and preserve our planet for future generations.

## Data Availability

This review cites previously published data.
